# Prediction of novel mouse TLR9 agonists using a random forest approach

**DOI:** 10.1186/s12860-019-0241-0

**Published:** 2019-12-20

**Authors:** Varun Khanna, Lei Li, Johnson Fung, Shoba Ranganathan, Nikolai Petrovsky

**Affiliations:** 1 College of Medicine and Public Health, Flinders University, Adelaide, SA 5042 Australia; 2 Vaxine Pty Ltd, 11 Walkley Avenue, Warradale, Adelaide, SA 5042 Australia; 3 Department of Molecular Sciences, Macquarie University, Sydney, NSW 2109 Australia

**Keywords:** Toll-like receptor 9, CpG, Machine learning, Random Forest, CpG, Oligonucleotides, Imbalanced data

## Abstract

**Background:**

Toll-like receptor 9 is a key innate immune receptor involved in detecting infectious diseases and cancer. TLR9 activates the innate immune system following the recognition of single-stranded DNA oligonucleotides (ODN) containing unmethylated cytosine-guanine (CpG) motifs. Due to the considerable number of rotatable bonds in ODNs, high-throughput in silico screening for potential TLR9 activity via traditional structure-based virtual screening approaches of CpG ODNs is challenging. In the current study, we present a machine learning based method for predicting novel mouse TLR9 (mTLR9) agonists based on features including count and position of motifs, the distance between the motifs and graphically derived features such as the radius of gyration and moment of Inertia. We employed an in-house experimentally validated dataset of 396 single-stranded synthetic ODNs, to compare the results of five machine learning algorithms. Since the dataset was highly imbalanced, we used an ensemble learning approach based on repeated random down-sampling.

**Results:**

Using in-house experimental TLR9 activity data we found that random forest algorithm outperformed other algorithms for our dataset for TLR9 activity prediction. Therefore, we developed a cross-validated ensemble classifier of 20 random forest models. The average Matthews correlation coefficient and balanced accuracy of our ensemble classifier in test samples was 0.61 and 80.0%, respectively, with the maximum balanced accuracy and Matthews correlation coefficient of 87.0% and 0.75, respectively. We confirmed common sequence motifs including ‘CC’, ‘GG’,‘AG’, ‘CCCG’ and ‘CGGC’ were overrepresented in mTLR9 agonists. Predictions on 6000 randomly generated ODNs were ranked and the top 100 ODNs were synthesized and experimentally tested for activity in a mTLR9 reporter cell assay, with 91 of the 100 selected ODNs showing high activity, confirming the accuracy of the model in predicting mTLR9 activity.

**Conclusion:**

We combined repeated random *down-sampling* with random forest to overcome the class imbalance problem and achieved promising results. Overall, we showed that the random forest algorithm outperformed other machine learning algorithms including support vector machines, shrinkage discriminant analysis, gradient boosting machine and neural networks. Due to its predictive performance and simplicity, the random forest technique is a useful method for prediction of mTLR9 ODN agonists.

## Background

Toll-like receptors (TLRs) represent an ancient evolutionary host immune defense system. There are 13 expressed TLR genes in mice (10 in humans), and each is devoted to recognizing a distinct set of pathogen associated molecular patterns (PAMPs) that are not found in healthy vertebrate cells, making them an important tool to help fight infections [1]. TLRs 1, 2, 4, 5 and 6 are extracellular and are situated in the plasma membrane where they bind bacterial cell wall components such as lipoteichoic acids, lipopolysaccharides, lipoproteins, and flagella. TLRs 3, 7, 8, 9 are located in endosomes where they recognize specific nucleic acid sequences expressed by various pathogens [2]. The extracellular signaling domain of TLR9 forms a horseshoe shaped dimer that forms a sandwich that clasps two CpG oligonucleotides (ODN) resulting in the cytoplasmic domains coming into close proximity thereby triggering downstream signaling [2]. Upon activation, TLR9 triggers an innate immune response characterized by the production of pro-inflammatory cytokines such as TNF-α, IL-1, IL-6, and IL-12.

Some synthetic single-stranded ODNs that contain unmethylated CpG motifs mimic bacterial DNA and can bind and activate TLR9 leading to cytokine secretion and enhancement of adaptive immune responses. Synthetic TLR9-active ODNs have shown utility as vaccine adjuvants and anti-cancer immunotherapeutic agents. To identify a good TLR9 ligand, typically a large library of ODNs needs to be synthesized and screened on cell lines, which is a time consuming and expensive task. We hypothesized that modern in silico high-throughput screening (HTS) methods may improve the ability to identify novel highly active TLR9 ligands. In silico screening, also known as virtual screening (VS), has been widely used to enrich datasets with compounds that have a higher probability of binding to the target of interest [3–5], and has an advantage over traditional screening or physical HTS due to its massively parallel processing ability; hence millions of compounds can be assessed economically in parallel. This is particularly important when the search space for potential ODNs TLR9 ligands is taken into consideration. A typical single-stranded ODN TLR9 agonist is 24 nucleotides in length, which amounts to 4^24^ total number of possible ODNs.

VS methods are of two major classes based on the availability of structural information. If the 3D structure of a receptor is known, structure-based virtual screening (SBVS) [6] techniques such as homology modeling, molecular docking and molecular dynamics can be used. However, if the structural information of the receptor is lacking, then ligand-based virtual screening (LBVS) [7] techniques such as quantitative structure-activity relationship and machine learning are more appropriate. SBVS involves molecular complex optimization to find the most favorable 3D binding conformation of the ligand. Consequently, SBVS is unsuitable for high-throughput screening of ligands like 24-mer ODNs, which have over 100 rotatable bonds. On the other hand, LBVS is computationally inexpensive, easy to use and might therefore be useful in the screening of TLR9 activating ODNs.

In a recent review, Murgueitio et al. [8] discussed the use of various computational approaches to investigate the structure and function of TLR receptors. To discover potential TLR ligands. Zatsepin et al. [9] screened a library of 1.8 million commercially available compounds to discover TLR9 antagonists by using computational chemistry and cell-based assays. The authors reported 21 potential TLR9 antagonists with IC50 lower than 10 μM, with five of them having IC50 values below 1 μM. Zhou et al. [10] constructed a 3D structure of human TLR9 ectodomains, complexed with CpG ODNs using homology modeling, then used molecular docking to study the interactions between TLR9 and the ODNs. They reported that leucine rich region (LRR)-11 was the main region in TLR9 responsible for ODN binding. The authors further reported that five positively charged residues within LRR11 were specifically involved in the ODN binding to TLR9. Nagpal et al. [11] reported a support vector machine model to predict ODNs with TLR9 activity with the model achieving a maximum Matthews Correlation Coefficient of 0.75 with an accuracy of 87%.

TLR9 ligand prediction tools require availability of well-annotated ODN datasets with experimentally determined TLR9 activity data. Machine learning (ML) based techniques such as decision trees, random forest, support vector machines and neural networks can then be applied to such ODN datasets. ML is an umbrella term for statistical models built to discover patterns in existing data to explain unseen data. ML models are very powerful tools that have been used in the past to predict and classify the pharmacokinetics or toxicological profiles of compounds [12], predict biological activities or toxicity [13] and assist in screening and optimization of compounds [5].

To our knowledge, this is the first report on the use of random forest-based approaches to predict novel mTLR9 ligands based on an in-house experimentally validated ODN dataset, with 91% prediction accuracy shown by experimental validation.

## Results

The main goal of this study was to build a ML model that could distinguish ODNs that have high activity for mTLR9 from ODNs with low activity. We used 117 ODNs with known high mTLR9 activity, as positive examples while 274 ODNs with low activity were used as negative examples.

### Motif analysis

**Fig. 1:**
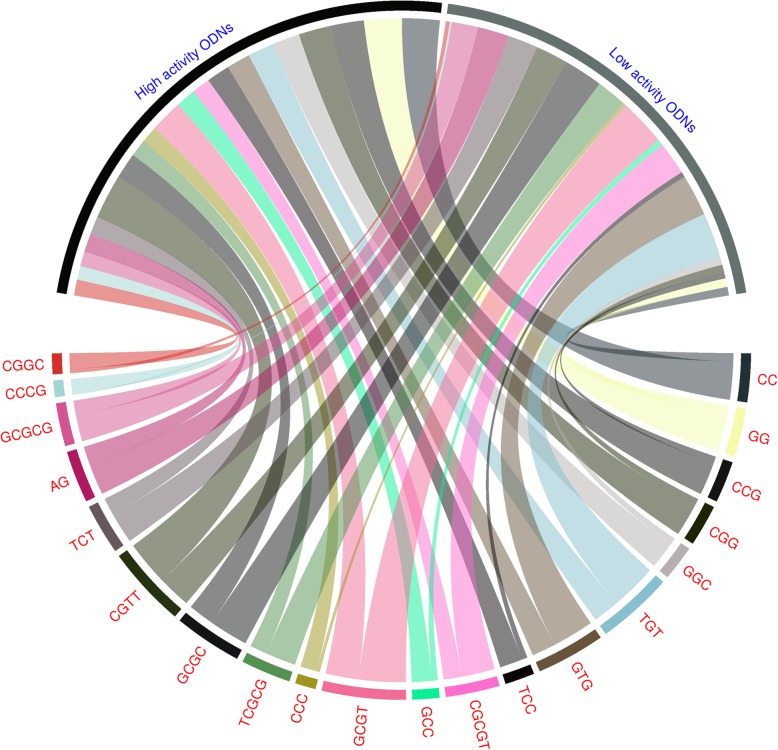
Top 20 motifs in mTLR9 active ODN arranged in clockwise manner based on the absolute difference in the percentage of occurrence in high and low activity groups of ODNs. The width of the ribbon shows average percent composition of the motifs in each group

We first analysed the dataset to understand the occurrence of sequence motifs in mTLR9 activating ODNs. We observed an uneven distribution of motifs with a few motifs such as ‘GG’ or ‘CC’ present in 57% of the ODNs in the high activity group compared to only 13% of the ODNs in the low activity group. Figure 1 shows the percentage of ODNs in the top 20 motifs arrange in a clockwise manner, based on the absolute difference in the percentage of occurrence in high and low mTLR9 activity groups of ODNs. All motifs having an absolute difference above 10% are shown in Additional file 1.

**Fig. 2:**
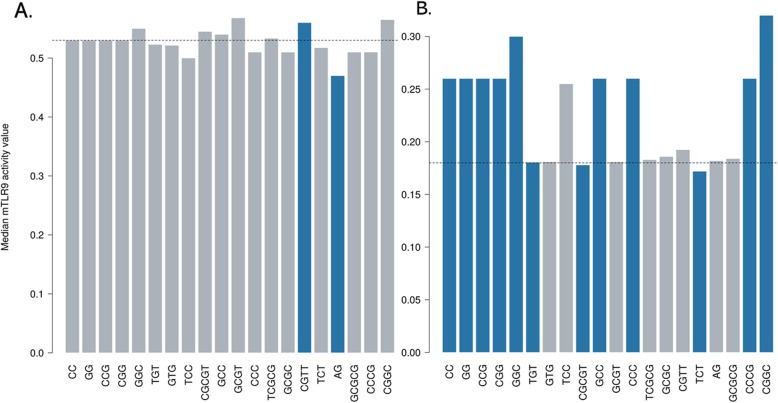
The effect of top 20 motifs in the high (**a**) and low (**b**) mTLR9 activity group of ODNs in the dataset. The darker bars represent a significant difference in the median mTLR9 activity score due to the presence of motif in the ODNs. The dotted line shows the median mTLR9 activity of 0.53 and 0.18 for the ODNs in the high and low activity groups, respectively, in the dataset

We further analyzed the effect of motif occurrence on the mTLR9 activity score in the high and low activity groups of ODNs in the dataset. Using the Mann-Whitney U test we compared the median mTLR9 activity score of ODNs with a motif to those without the motif for the two classes and calculated the *p* values. The significance threshold was set at 0.05. Figure 2 shows the effect of top 20 motifs occurrence in high (Fig. 2a) and low (Fig. 2b) mTLR9 active group of ODNs. The darker colored bars stand for a significant difference in the median mTRL9 activity score (*p* < 0.05) due to the presence of the motif in the ODNs. The dotted line is the median mTLR9 score of 0.53 and 0.18 for the high and low activity groups of ODNs, respectively. Within the low activity group (Additional file 2), we found that presence of motifs such as ‘CC’, ‘GG’, ‘GGC’, ‘GCC’, ‘CCCG’ and ‘CGGC’ significantly increases the median mTLR9 activity score, while the presence of motifs e.g. ‘TGT’, ‘CGCGT’ and ‘TCT’ further lowers the activity of ODNs. In contrast, we found presence of ‘CGTT’ motif to significantly improve while ‘AG’ motif to significantly decrease the median mTLR9 activity score of the ODNs in the high activity group (Additional file 3). Since there was no single motif that could account for the mTLR9 activity score of the ODNs, we surmised that the combination of motifs and their interaction with the TLR9 receptor was responsible for determining overall mTLR9 activity.

### Model selection

**Fig. 3:**
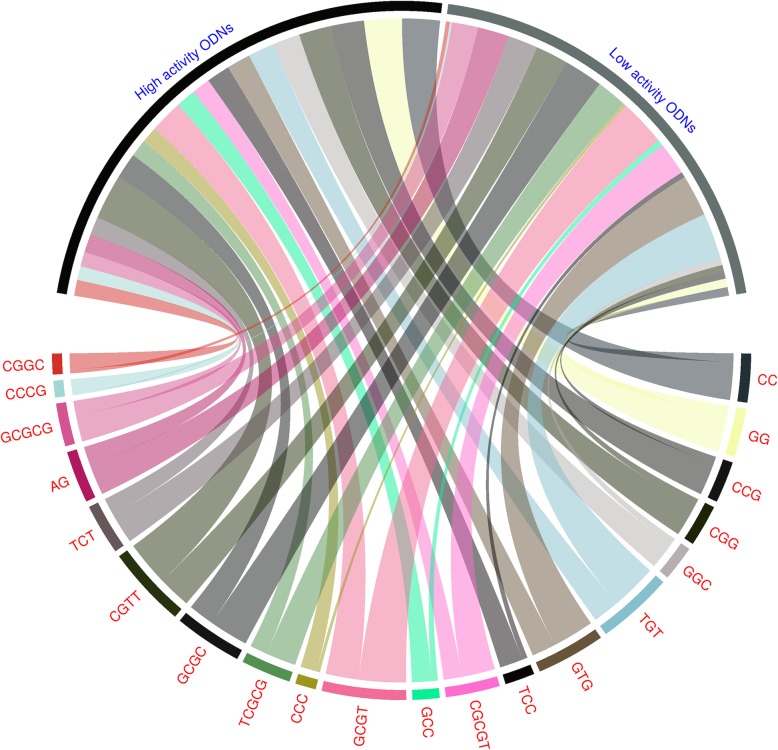
Mean and standard deviation of Balanced Accuracy rates of the five classifiers on the twenty bootstrap test samples using k-fold cross-validation scheme. Mean balanced accuracy rate of RF model was greater than all five algorithms in all the folds

Mean classification levels achieved by all algorithms in different *k*-fold cross validation schemes when applied to 20 bootstrap test samples obtained using the *down-sampling* technique are shown in Fig. 3. We found that overall RF model either outperformed or was on par with the other prediction algorithms in all four cross validation schemes. In five-fold cross validation the best rates were achieved by the RF and SVM model with a maximum balanced accuracy of 95.65% and mcc of 0.91 (Additional file 4). The mean balanced accuracy and mean MCC for RF model in five-fold cross validation was 77.8% and 0.57, respectively, with standard deviations of 0.08 and 0.15, respectively (Table 1). In ten-fold cross validation, RF and GBM achieved the best results with the maximum balanced accuracy and mcc of 89.13% and 0.78, respectively (Additional file 5). The mean balanced accuracy and mcc for the RF model in ten-fold cross validation was 78.9% and 0.60, respectively, with standard deviations of 0.06 and 0.11, respectively (Table 1). In 15-fold cross validation the best results were achieved by RF and SVM with the maximum balanced accuracy and mcc of 86.9% and 0.74, respectively (Additional file 6). The mean balanced accuracy and mcc for the RF model in 15-fold was 77.0% and 0.55, respectively with standard deviations of 0.06 and 0.11, respectively (Table 1). In 20-fold cross validation random forest achieved the best result with the maximum balanced accuracy and mcc of 87.0% and 0.75, respectively (Additional file 7). The mean balanced accuracy and mcc of RF model was 79.7% and 0.61, respectively, with standard deviations of 0.05 and 0.09, respectively (Table 1). Overall, the RF algorithm outperformed in all other ML methods, for different cross-validation values. We therefore selected RF with the 20-fold cross-validation scheme, having maximum mean balanced accuracy and MCC and minimum standard deviation on both measures, to perform the mTLR9 activity predictions for the randomly generated ODN dataset.

**Table 1:** Mean and standard deviation (SD) values of the balanced accuracy and Matthews Correlation Coefficient (MCC) for all five learning algorithms in 20 bootstrap test samples. The best values in each fold category are underlined with the overall best in bold

Algorithm	Cross-validation	Mean balanced accuracy	SD balanced accuracy	Mean MCC	SD MCC
RF	5-fold	77.8%	0.08	0.57	0.15
GBM	5-fold	76.8%	0.07	0.55	0.12
SDA	5-fold	74.6%	0.08	0.50	0.14
SVM	5-fold	77.1%	0.08	0.55	0.16
NN	5-fold	74.1%	0.07	0.50	0.13
RF	10-fold	78.9%	0.06	0.60	0.11
GBM	10-fold	77.7%	0.05	0.57	0.10
SDA	10-fold	75.8%	0.06	0.53	0.11
SVM	10-fold	78.4%	0.05	0.58	0.11
NN	10-fold	72.9%	0.05	0.48	0.10
RF	15-fold	77.0%	0.06	0.55	0.11
GBM	15-fold	76.9%	0.06	0.55	0.11
SDA	15-fold	73.5%	0.06	0.49	0.11
SVM	15-fold	76.3%	0.05	0.53	0.11
NN	15-fold	72.6%	0.07	0.47	0.15
**RF**	**20-fold**	**79.7%**	**0.05**	**0.61**	**0.09**
GBM	20-fold	78.5%	0.07	0.58	0.12
SDA	20-fold	76.1%	0.08	0.54	0.14
SVM	20-fold	75.4%	0.05	0.52	0.09
NN	20-fold	74.9%	0.07	0.52	0.13

### External validation

**Fig. 4:**
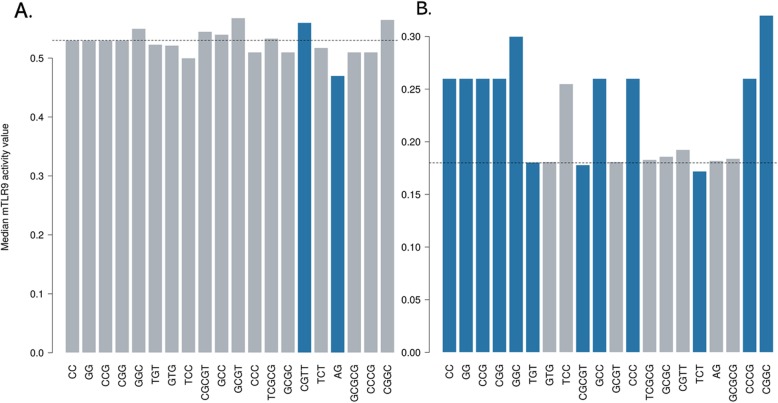
Measured mTRL9 activity values of the 100 top predicted TLR9 active ODNs. The dotted black line is the cutoff value for the ODNs in the high activity group used in building the model

External validation is the final step to evaluate the realistic performance of any prediction model. In this technique, the performance of the model is evaluated on a new dataset not used in training or testing the model. To rigorously evaluate the performance of our model, we randomly generated 6000 24-mer ODN sequences using an in-house written Python script and then screened and ranked these randomly generated ODN for mTLR9 activity using our RF model. These ODNs were not present in our original dataset of 396 ODNs used for model building or training, and as they were virtual we had no prior knowledge of their likely mTLR9 activity at the time of model prediction. Our RF model predicted 545 of these 6000 random ODNs to be of high activity and we selected the top 100 for chemical synthesis, and then experimental tested them for mTLR9 activity using the RAW-Blue reporter cell line that expresses mTLR. Ninety-one (91%) of the predicted high activity ODNs had a mTLR9 activity value above 0.4, confirming the high accuracy of the model in predicting ODN sequences with positive mTLR9 activity (Fig. 4). This demonstrates that our mTLR9-specific RF prediction model is rigorous, with a strong performance on making predictions on a completely independent dataset.

## Discussion

In this study we demonstrated the feasibility of using an RF model for in silico screening of synthetic ODNs to detect high activity mTLR9 agonists. Multiple sequence features such as simple counts of nucleotides, the distance between motifs and graphically derived features like the moment of inertia were calculated before building the RF model. We observed higher occurrence of several motifs such as ‘CGGC’, ‘CCCG’, ‘GCC’, ‘CGG’, ‘GGC’, ‘CCG’, ‘CCC’, ‘GG’ and ‘CC’ in high activity as compared to low activity ODNs. This means that these cytosine and guanine rich motifs along with the key unmethylated CpG dinucleotide contribute to strong mouse TLR9 activation. Interestingly, this is in contrast with the thymine rich motifs reported for TLR9 stimulatory ODNs by Nagpal et al. [11]. This may be due the fact that our ODN training set was mouse specific whereas the dataset used by Nagpal et al. [11] was not specific to any organism. On further analysis we found 15 and 4 motifs which significantly increased, or decreased, respectively, mTLR9 activity in the low activity group (Additional file 2), whereas, we found only 3 and 4 motifs in the high activity ODNs which significantly (*p* value < 0.05) increased or decreased, respectively, mTLR9 activity (Additional file 3). Furthermore, we discovered motifs which significantly decreased mTLR9 activity in both low and high groups. For example, ‘CGCGTG’ and sub motifs like ‘GCGTG’ and ‘CGCGT’, decreased the activity of ODNs in both the high and low groups. However, we were unable to identify motifs that increased mTLR9 activity for both groups of ODNs. This suggests that a combination of motifs might be required to increase activity of ODNs in the high group whereas the activity of low ODNs can be improved even by inclusion of a single motif. Co-occurrence of motifs and their effect on mouse TLR9 activity can be analyzed in the future to discover combinations of motifs responsible for the increase in the activity of ODNs in both groups.

The performance of the RF model was compared to other methods, which were trained on the same data. The average classification accuracy achieved by all the methods when applied to 20 bootstrap test samples in four different cross-validation schemes is shown in Fig. 3. The results demonstrated that the RF model had the superior performance on the test datasets in most of the scenarios. The GBM and SVM classifiers also had reasonable classification accuracy rates, however, RF outperformed them in 20-fold cross validation scheme.

The selected RF model on average correctly classified 79.1% of the ODNs in the training set with high activity for mTLR9 and 80.2% of ODNs with low activity. The RF thereby achieved an overall balanced accuracy of 79.7%.

Finally, the RF model was used to virtually screen 6000 randomly generated ODNs from which it predicted 545 ODNs to have high activity for mTLR9. Due to large number of predicted positive hits, the top 100 ODNs were selected for synthesis and testing for mTLR9 activity in vitro. Ninety one out of the 100 synthesized ODNs were found to have mTLR9 activity above the cutoff of 0.4 for high activity ODNs confirming the prediction potential of the RF model. However, Fig. 4 shows that the majority of predicted ligands had an activity value ranging from 0.5 to 0.7, which indicates that the model might need to be further fine-tuned to get even higher activity ligands, with a much larger dataset than the randomly generated 6000 oligonucleotides screened to find high activity ligands.

## Conclusions

In this study we found several sequence motifs that help explain the mTLR9 activity of CpG ODNs. Motifs including ‘CGTT’, ‘GGC’, ‘GCC’ and ‘CCCG’ significantly improved, whereas motifs such as ‘AG’, ‘TCT’ and ‘CGCGT’ significantly decreased, the activity of mTLR9 ODNs. Further, we developed and validated an RF model for predicting ODNs with mTLR9 activity. The results showed that the RF method was well suited for predicting high activity mTLR9 specific ODNs and outperformed various other learning algorithms such as SVM, SDA, NN and GBM. The model was used to screen a random library of 6000 ODNs and correctly identified 91 out of 100 ODNs that were subsequently confirmed to have mTLR9 activity. This shows the power of machine learning models for discovering novel TLR9 agonists. The lead mTLR9 active ODN candidates from the above studies are now being tested as vaccine adjuvants and anti-cancer agents in relevant mouse models.

## Materials and methods

### Preparation of the dataset

**Fig. 5:**
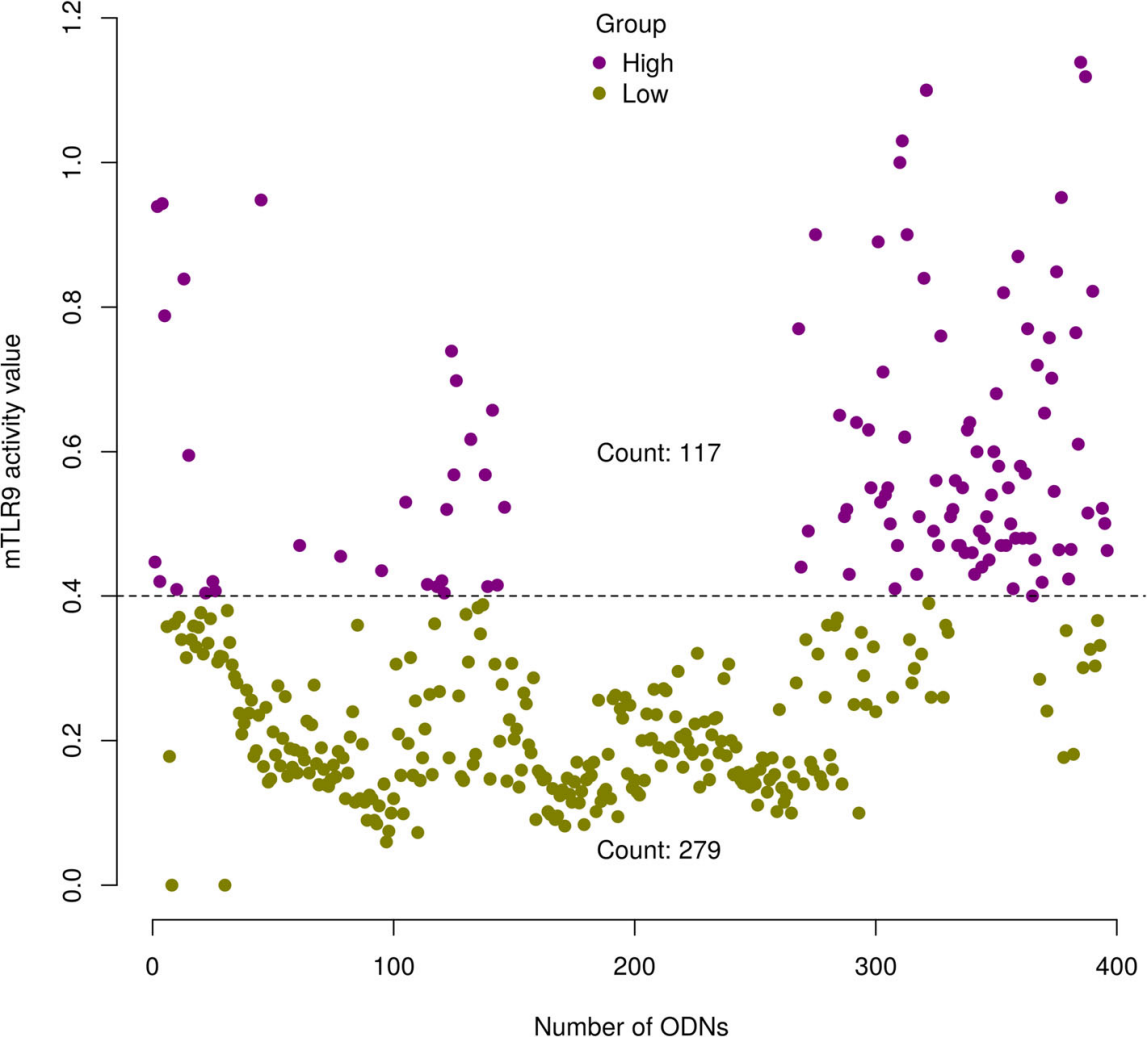
The measured mTLR9 activity value of all the synthesized 24-mer ODNs in the dataset. The ODNs were divided into two groups of high (shown in purple) and low (shown in green) activity using a cutoff score of 0.4, based on the optimal density (OD) results from the Raw-blue reporter cell assay

The quality of the training dataset determines the quality of the resulting machine learning model. Missing or insufficient data, mislabeling of the target variable, and irrelevant features may complicate the learning task and hinder the performance of the trained model. The sequences of ODNs with experimentally determined mTLR9 activity were obtained from in-house data we generated on synthesized ODNs that were characterized using a mouse TLR9 expressing reporter cell line (RAW-Blue cells, Invivogen, USA). The dataset consisted of 396 ODNs with mTLR9 activity values ranging from 0.0 (no activity) to 1.14 (high activity). The ODNs were grouped into two classes (Fig. 5) based on their respective activity value (i.e. 0.4 and above: high activity and below 0.4: low activity), resulting in a high activity group (count 117) and a low activity group (count 279). To ensure data quality, it is customary to check and remove any outliers, impute the missing data, check, and assign the variables the correct datatype. Our dataset had neither missing values nor outliers and therefore, no further action was required in cleaning the dataset. However, to avoid overtraining the model with similar ODNs, the diversity of the dataset was increased by limiting the similarity within the group. This was achieved by clustering the ODNs within a group using the binary fingerprint features we developed during this study and applying a clustering cutoff of 0.85 to remove similar ODNs. This resulted in the removal of five ODNs from the low activity group with 274 remaining. All ODNs in the high group (count 117) were dissimilar enough not to breach the similarity cutoff and were retained.

**Fig. 6:**
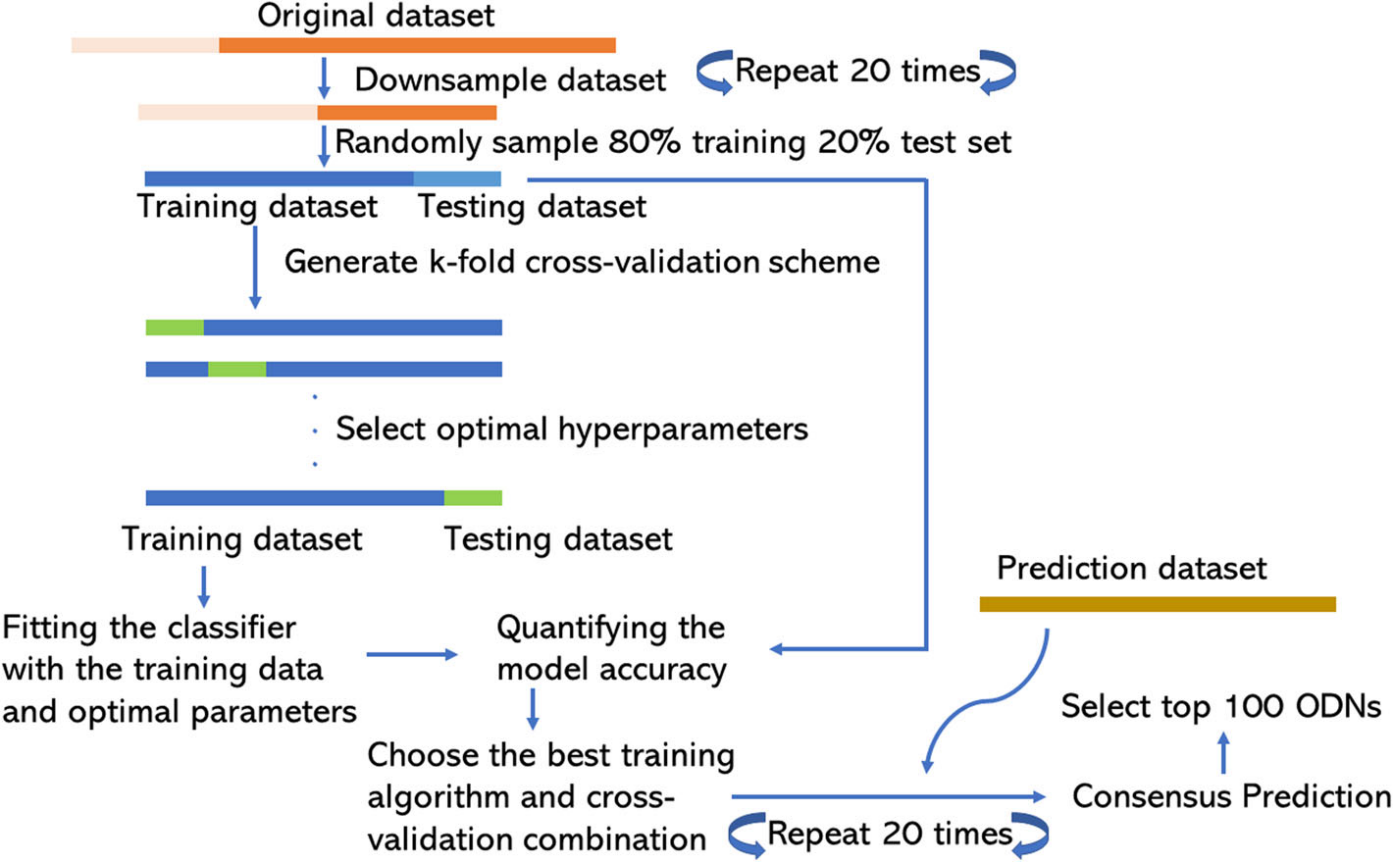
Flowchart of methodology adopted

In our training dataset, the number of ODNs with low mTLR9 activity was approximately 2.5 times more than the number of ODNs with high mTLR9 activity. Therefore, we used the *down-sampling* technique to balance the dataset, so that 50% of the samples were derived from the set of ODNs with high activity and 50% from the set of ODNs with low activity. Subsequently, the *down-sampled* dataset was subdivided into training (80%) and testing (also known as validation) sets (20%), using a random sampling technique and the ODNs in the test set were excluded from model training. In order to choose the best classifier with *k*-fold cross validation, the performance of our models were measured using 20 *down-sampled* test sets. The overall methodology adopted in the study is shown in Fig. 6.

In Table 2, we present the composition of the dataset used in this study. For each instance, the training dataset was composed of 188 ODNs (derived from 94 ODNs with high and low mTLR9 activity each). The test dataset used to evaluate the performance of a model was composed of 46 ODNs (23 each from the two groups of high and low mTLR9 activity). For the prediction set, we used an in-house python script to randomly generate 6000 24-mer ODNs, to capture the diversity of the 24-mer CpG-ODN universe. Every ODN in the prediction set was classified using the selected model and cross-validation scheme in a loop. For the final prediction, a consensus of the 20 predictions were taken for each ODN in the prediction set. Finally, the top 100 high activity predicted ODNs were selected for synthesis and experimental testing using the RAW-Blue reporter cell line assay. The training and test set ODNs along with experimental activity information are available in Additional file 8.

**Table 2:** Composition of the training and test sets at any instance

Dataset	Training set	Testing set	Total
High	94	23	117
Low	94	23	117
Total	188	46	234
Prediction set	_	_	6000

### Molecular feature calculation and selection

It is possible to generate a large number of features for the ODN sequence data that can be used to construct machine learning models. However, there are several problems in using all the possible features as (i) some of the features may be highly correlated (ii) some may not be relevant and may contribute to the noise in the model and (iii) using a large number of features may lead to overfitting. Additionally, constructing models with many features is computationally demanding [14]. Therefore, one of the most important aspects of creating a good ML model is the choice of appropriate features that can help explain the behavior of interest based on Occam’s Razor principle (i.e. simple models are more likely to be closer to reality than complex models.) [15]. While there are a variety of features used in bioinformatics for sequence data, we used the binary fingerprint features and numerical features, including count and position of motifs, distance of the motifs with respect to the start position and graphically derived features such as the moment of inertia and radius of gyration, to train the model [16].

### Fingerprint features

To generate fingerprint features, a *fasta* formatted file containing all high activity ODN sequences was analysed using an in-house Perl subroutine, to chop each sequence into motifs of increasing length from two to six nucleotides and record the start positions of the motifs. For example, with a small hypothetical ODN ‘TCG’ of three nucleotides, two dinucleotides motifs TC1, CG2 and a trinucleotide TCG1 motif were generated. Finally, a dictionary of the motifs with at least 10% difference in the occurrence rate in low and high group of ODNs (count 67) was prepared. Subsequently, the dictionary was used to generate the binary fingerprint pattern for each sequence, where 1 showed the presence of a motif while 0 indicated its absence.

### Count of nucleotides

Different patterns of nucleotide usage in ODNs may lead to varied mTLR9 activity. Therefore, all nucleotide characters (A, T, G, C) were counted in a sequence and the Perl built-in dictionary data structure, *hash,* was used to store the count of each nucleotide. Ambiguous nucleotide characters or gaps were ignored if present.

### Calculating the distance between motifs with respect to their start positions

The most commonly occurring motifs were used to calculate the distance between motif features along with their specific location. To map the position of a motif in the ODNs, the sequence of each ODN was scanned for the presence of a motif and all the positions where each motif occurs were recorded. Using eqs. (1)–(3), the distance between the second and first, third and first and the third and second occurrence of the motifs were calculated for all the motifs.

1 $$ \boldsymbol{d}\_\boldsymbol{motif}\mathbf{2}\_\mathbf{1}=\boldsymbol{p}\mathbf{2}-\boldsymbol{p}\mathbf{1}+\boldsymbol{n} $$

2 $$ d\_ motif3\_1=p3-p1+n $$

3 $$ d\_ motif3\_2=p3-p2+n $$

where *d_motif* is the distance, p3, p2 and p1 are the position 3, position 2 and position 1 of the motif respectively, and ‘n’ is the number of nucleotides before the latter motif. In the case of the absence of a motif, 0 was substituted in the equation. It is important to keep ‘n’ in the equation to provide the specific location of the motifs within an ODN, because the calculated distance between motifs could be same in several ODNs. For example, in a sequence S1 = TATG**CG**TT**CG**TACTTGATCTGAC, the distance between CG motifs is 9–5 = 4. Similarly, for another sequence S2 = TGCTTTCTTGT**CG**TG**CG**GGCTGT, the distance between the CG motifs is 16–12 = 4, again. However, the descriptor *d_CG2_1* value for S1 and S2 are 12 and 19, respectively, with the addition of *n* to the simple distance formula of *d_motif*.

### Graphically derived features

The graphical representation of DNA sequences have been used for many applications including assessing phylogenetic relationships [17], characterization of neuraminidase gene in H5N1 avian flu [18] and for describing similarity/dissimilarity of DNA sequences [4]. In order to derive features, the 24-mer ODN sequences were represented as a 2D-graph, as previously described [16]. Briefly, each base in the sequence is represented as a material point on the graph which is treated as a rigid body and follows the rules of Newtonian dynamics. Numerical features such as the center of mass (μ_x_*,* μ_y_), the principal moment of inertia(*I*_*11,*_ *I*_*22*_) and radius of gyration (R_g_) were calculated for each sequence as described in [16].

### Feature selection

There are several feature selection methods used in machine learning to remove redundant or irrelevant features. These can be broadly divided into filter methods (e.g. correlation matrix, information gain, Chi-square score, principal component analysis, regression coefficients, variable importance) and wrapper methods (e.g. forward/backward selection, randomized methods that combine PLS with the genetic algorithm or Monte Carlo algorithm) [19–21]. Filter methods are easy to implement because there is no learning involved and depend only on the application of a cut-off value to reject features due to the low importance in the model construction. In the wrapper methods, the performance of a learning algorithm is evaluated to select the optimum subset of features therefore, it is a very computationally expensive process [19] and is best suited for a limited number of features. Furthermore, filter methods work well for text mining [19], and are applicable for ODN features, which are essentially nucleotide “words.”

Due to the large number of fingerprint features available (67 in total), we first filtered out the constant and near-constant features (features with < 0.3 standard deviation) also known as zero and near zero variance features using the *caret* package in R. Constant or near constant features take a unique value across samples and are uninformative. This resulted in the removal of 26 features. Since these features are binary in nature, we also checked and removed any linear combinations of features if present. This resulted in the removal of 31 features. To understand the distribution in the high and low group of ODNs we created a Cricos plot using the *circlize* package in R [22]. For all numerical features in addition to removing zero and near zero variance features we also calculated the correlation matrix and filtered out features that were highly correlated. The correlation coefficient was set at 0.85 and features with correlation above the cutoff value were removed. We then normalized the remaining features using centering and scaling techniques to make them unit independent. Subsequently, we merged the fingerprint and numerical features to give us a merged set of 40 features, listed in Table 3.

**Table 3:** Features used in this study

S.no	Feature	Description	Type
1	A	Count of A nucleotides	Numerical
2	T	Count of T nucleotides	Numerical
3	G	Count of G nucleotides	Numerical
4	C	Count of C nucleotides	Numerical
5	d_CG2_1	Distance between occurrences 2 and 1 of CG motif	Numerical
6	d_CG3_1	Distance between occurrences 3 and 1 of CG motif	Numerical
7	d_CG3_2	Distance between occurrences 3 and 2 of CG motif	Numerical
8	d_AG2_1	Distance between occurrences 2 and 1 of AG motif	Numerical
9	d_AG3_1	Distance between occurrences 3 and 1 of AG motif	Numerical
10	d_AG3_2	Distance between occurrences 3 and 2 of AG motif	Numerical
11	d_GG2_1	Distance between occurrences 2 and 1 of GG motif	Numerical
12	d_GG3_1	Distance between occurrences 3 and 1 of GG motif	Numerical
13	d_GG3_2	Distance between occurrences 3 and 2 of GG motif	Numerical
14	d_CC2_1	Distance between occurrences 2 and 1 of CC motif	Numerical
15	d_CC3_1	Distance between occurrences 3 and 1 of CC motif	Numerical
16	d_CC3_2	Distance between occurrences 3 and 2 of CC motif	Numerical
17	d_TCT2_1	Distance between occurrences 2 and 1 of TCT motif	Numerical
18	d_TCT3_1	Distance between occurrences 3 and 1 of TCT motif	Numerical
19	d_TCT3_2	Distance between occurrences 3 and 2 of TCT motif	Numerical
20	d_TTC2_1	Distance between occurrences 2 and 1 of TTC motif	Numerical
21	d_TTC3_1	Distance between occurrences 3 and 1 of TTC motif	Numerical
22	d_TTC3_2	Distance between occurrences 3 and 2 of TTC motif	Numerical
23	d_TGT2_1	Distance between occurrences 2 and 1 of TGT motif	Numerical
24	d_TGT3_1	Distance between occurrences 3 and 1 of TGT motif	Numerical
25	d_TGT3_2	Distance between occurrences 3 and 2 of TGT motif	Numerical
26	PMI1	Principal Moment of Inertia 1	Numerical
27	PMI2	Principal Moment of Inertia 2	Numerical
28	Mu_x	Center of mass in x direction	Numerical
29	Mu_y	Center of mass in y direction	Numerical
30	CG1	Presence of CG at position 1	Fingerprint
31	GC1	Presence of GC at position 1	Fingerprint
32	GT1	Presence of GT at position 1	Fingerprint
33	GT18	Presence of GT at position 18	Fingerprint
34	GCG6	Presence of GCG at position 6	Fingerprint
35	GT22	Presence of GT at position 22	Fingerprint
36	GT21	Presence of GT at position 21	Fingerprint
37	CGCG5	Presence of CGCG at position 5	Fingerprint
38	GC5	Presence of GC at position 5	Fingerprint
39	GT12	Presence of GT at position 12	Fingerprint
40	TC9	Presence of TC at position 9	Fingerprint

### Learning algorithms

In the current study, five ML algorithms, i.e. random forest, gradient boosting machine, shrinkage discriminant analysis, support vector machine and neural network were compared, and the best performing model was chosen for the prediction of novel mTLR9 active ODNs. To have a non-biased assessment of the performance, *k*-fold cross-validation was followed where one instance of the *down-sampled* training data was further divided into *k* partitions. The value of *k* varies from 5, 10, 15 to 20. For each partition, ODNs not included in the training were considered part of the testing dataset. Finally, the testing data of the instance was used to evaluate the classification accuracy of the model, with the best model selected for prediction on an independent validation dataset. A graphic representation of the general procedure is given in Fig. 6.

### Random Forest algorithm

The Random Forest (RF) algorithm was introduced by Breiman in 2001 [23] and is one of the most powerful ensemble machine learning technique that make predictions by averaging over several independent base learners in order to identify the class label for unknown instances. The base learners are usually the Classification and Regression Trees (CART) constructed using a sample with replacement from the training data with the controlled variation. RF can be used for both classification and regression tasks. It can manage missing values, outliers efficiently and perform well with imbalanced datasets. The detailed account of RF methodology is present in the literature [23, 24]. Briefly RF takes advantage of two powerful statistical techniques, bagging and random feature selection. In bagging each tree is trained on a bootstrap sample (sampling with replacement) and the predictions are made by the majority vote of the trees. Furthermore, in RF instead of using all the features, RF randomly selects a set of features to split at each node when growing a tree. To assess the performance of the RF algorithm, RF performs a type of cross-validation using the out-of-bag (OOB) samples (samples which are not included in the training set). The concept of variable importance is inbuilt in the RF algorithm and the importance is measured by the Gini impurity criterion index [25]. We used the *caret* package in R to evaluate the performance and developed an ensemble of 20 different RF models for final prediction. The *mtry* parameter was tuned using the *tuneGrid* argument in the train function.

### Performance metrics

The accuracy of the five ML algorithms was measured by presenting the prediction results in the form of a confusion matrix and the variety of performance measures were calculated based on the following statistical measures:

- TP, true positives – the total number of correctly classified high activity ODNs.
- TN, true negatives – the total number of correctly classified low activity ODNs.
- FP, false positives – the total number of low activity ODNs incorrectly classified as high activity ODNs.
- FN, false negatives – the total number of high activity ODNs incorrectly classified as low activity ODNs.

Using the measures above, a series of statistical metrics were computed including sensitivity (Se), specificity (Sp), Balanced Accuracy (Ba), Matthews correlation coefficient (MCC) and precision.

The recall rate for the members of the positive class (high activity ODNs) is given by sensitivity, in eq. (4):

4 $$ senstivity=\frac{TP}{TP+ FN} $$

Similarly, the recall rate for the members of the negative class (low activity ODNs) is given by specificity, in eq. (5):

5 $$ specificity=\frac{TN}{TN+ FP} $$

The balanced accuracy of the model was calculated based on the eq. (6):

6 $$ balanced\ accuracy=\frac{senstivity+ specificity}{2} $$

We then calculated the MCC from eq. (7); the coefficient returns a value between + 1 and − 1. The higher the value of the coefficient, the better the classification result.

7 $$ mcc=\frac{\left( TP\ast TN\right)-\left( FP\ast FN\right)}{\sqrt{\left( TP+ FP\right)\left( TP+ FN\right)\left( TN+ FP\right)\left( TN+ FN\right)}} $$

Finally, the precision was computed to measure the reproducibility of the results, in eq. (8):

8 $$ precision=\frac{TP}{TP+ FP} $$

### Mouse RAW-blue TLR9 reporter cell assay

RAW-Blue™ cells are derived from the murine RAW 264.7 macrophage cell line with chromosomal integration of a secreted embryonic alkaline phosphatase (SEAP) reporter construct inducible by NF-κB and AP-1 and were acquired from InvivoGen. The presence of agonists of mouse TLR9 activates downstream signaling pathways leading to the activation of NF-κB and AP-1, and the subsequent secretion by the RAW cells of SEAP. Levels of SEAP in the culture supernatant are measured chromatographically using the detection medium QUANTI-Blue™. RAW-Blue cells were cultured in DMEM supplemented with 10% (v/v) heat-inactivated fetal bovine serum, penicillin-streptomycin 10,000 U/mL (Gibco), and Normocin 100 μg/mL (InvivoGen). Subsequently, RAW-Blue cells were seeded at a density of approximately 1 × 105 cells/well in a volume of 180 μL/well in a flat-bottom 96-well culture plate (Greiner-One). ODNs were diluted in saline and added to the culture plate containing RAW-Blue cells to the total volume of 200 μL. After culturing the cells for 3 h, the levels of SEAP were determined in the supernatant with QUANTI-Blue™ Solution (InvivoGen) by reading the absorbance at wavelength of 650 nm.

## Supplementary information

**Additional file 1.** Sequence motifs in mTLR9 active ODNs having an absolute difference in the occurrences above 10% in high and low activity groups of ODNs, arranged in a clockwise manner. The width of the ribbon shows the average percentage composition of the motifs each group.

**Additional file 2.** The effect of ODN motif occurrences on the median mTLR9 activity in the low activity group. The median RAW-Blue activity for all the ODNs in the low activity group was 0.18. Increase or decrease in the median activity values due to the presence of a motif are coloured green and red, respectively, with statistically significant values in bold. The significance threshold was set at *p*-value < 0.05. The motifs are arranged in alphabetical order.

**Additional file 3.** The effect of ODN motif occurrences on the median mTLR9 activity in the high activity group. The median RAW-Blue activity for all the ODNs in the high activity group was 0.53. Increase or decrease in the median activity values due to the presence of a motif are coloured in green and red, respectively, with statistically significant values in bold. The significance threshold was set at *p* value < 0.05. The motifs are arranged in alphabetical order.

**Additional file 4.** Results of five-fold cross-validation.

**Additional file 5.** Results of ten-fold cross-validation.

**Additional file 6.** Results of fifteen-fold cross-validation.

**Additional file 7.** Results of twenty-fold cross-validation.

**Additional file 8.** ODNs used as test and training sets for building the prediction model, along with activity information.

## Data Availability

All data reported in this study are available as Tables and Supplementary data. The cell line used in the assay is commercially available from Invivogen Inc. [26].

## Abbreviations

Ba: Balanced Accuracy
CART: Classification and Regression Trees
FN: False negatives
FP: False positives
GBM: Gradient Boosting Machine
HTS: High-throughput screening
LBVS: Ligand-based virtual screening
MCC: Matthews correlation coefficient
ML: Machine learning
mTLR9: Mouse Toll-like receptor 9
NN: Neural Network
OBB: Out-of-bag
ODN: Oligodeoxynucleotides
PAMPs: Pathogen associated molecular patterns
RF: Random Forest
SBVS: Structure-based virtual screening
SDA: Shrinkage discriminant analysis
SEAP: Secreted embryonic alkaline phosphatase
SVM: Support Vector Machine
TLR9: Toll-like receptor 9
TN: True negatives
TP: True positives
VS: Virtual screening
