# Supplementary material for: Prediction of novel mouse TLR9 agonists using a random forest approach
Source: BMC Mol Cell Biol. 2019 Dec 20;20(Suppl 2):56. doi: 10.1186/s12860-019-0241-0 (PMC6924143; doi:10.1186/s12860-019-0241-0)
Supplement: Supplementary file 1 — Additional file 1. Sequence motifs in mTLR9 active ODNs having an absolute difference in the occurrences above 10% in high and low activity groups of ODNs, arranged in a clockwise manner. The width of the ribbon shows the average percentage composition of the motifs each group. [file 12860_2019_241_MOESM1_ESM.pdf]

Prediction of novel mouse TLR9 agonists using a random forest approach

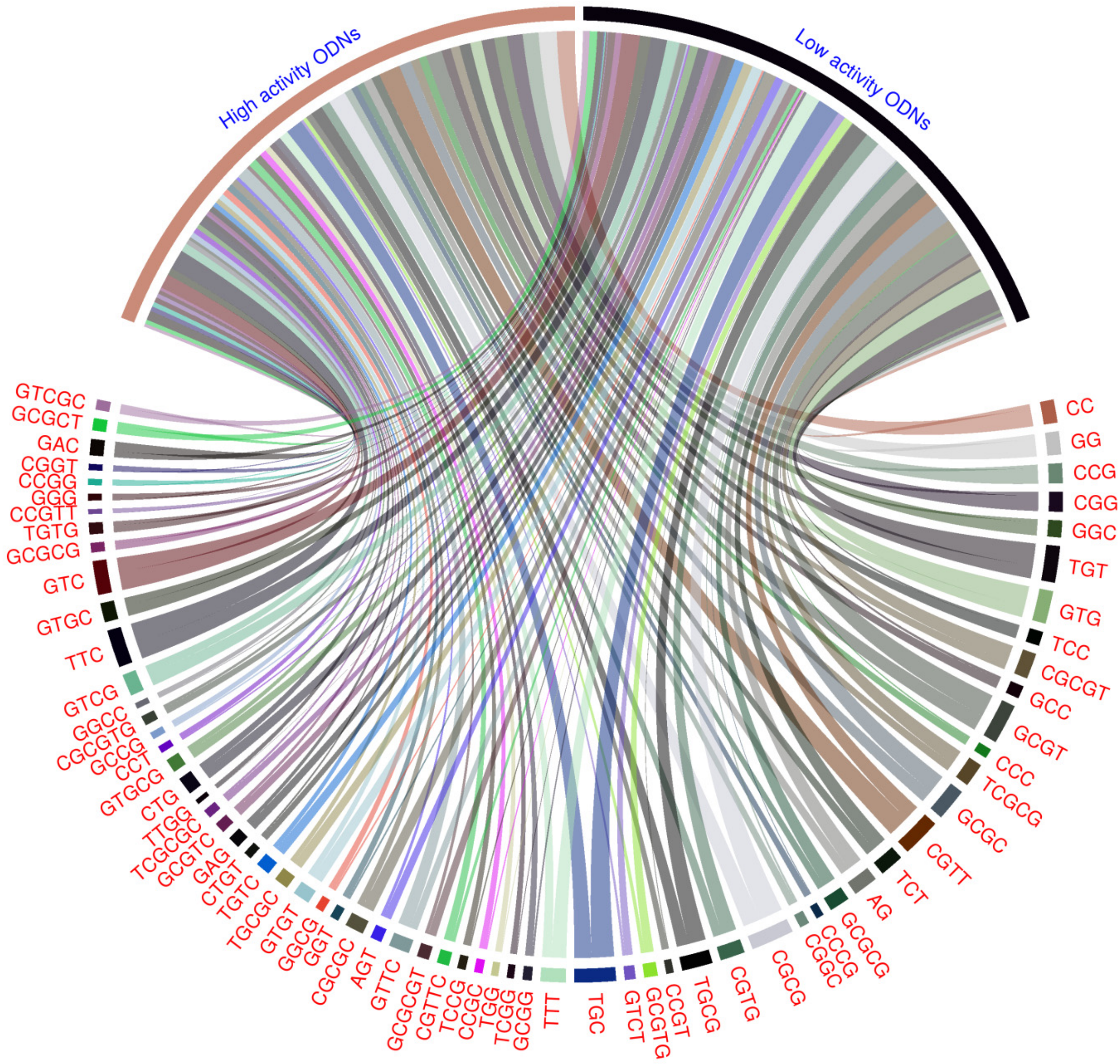

Additional File 1: Sequence motifs in mTLR9 active ODNs having an absolute difference in the occurrence above 10% in high and low activity groups of ODNs, arranged in a clockwise manner. The width of the ribbon shows the average percentage composition of the motifs in each group.
