# Supplementary material for: Prediction of novel mouse TLR9 agonists using a random forest approach
Source: BMC Mol Cell Biol. 2019 Dec 20;20(Suppl 2):56. doi: 10.1186/s12860-019-0241-0 (PMC6924143; doi:10.1186/s12860-019-0241-0)
Supplement: Supplementary file 2 — Additional file 2. The effect of ODN motif occurrences on the median mTLR9 activity in the low activity group. The median RAW-Blue activity for all the ODNs in the low activity group was 0.18. Increase or decrease in the median activity values due to the presence of a motif are coloured green and red, respectively, with statistically significant values in bold. The significance threshold was set at p-value < 0.05. The motifs are arranged in alphabetical order. [file 12860_2019_241_MOESM2_ESM.pdf]

Prediction of novel mouse TLR9 agonists using a random forest approach

Additional File 2: The effect on the median mTLR9 activity on ODNs in the low activity group due to the presence or absence of a motif. The median mTLR9 activity for all the ODNs in the low activity group was 0.18. Increase or decrease in the median activity values due to the presence of a motif are coloured green and red, respectively, with statistically significant values in bold. The significance threshold was set at p value < 0.05. The motifs are arranged in the alphabetical order.

S.No	Motif	Median mTLR9 activity of ODNs with the motif	Median mTLR9 activity of ODNs without the motif	*p*-value
1.	AG	0.18	0.19	0.8800
2.	AGT	0.18	0.19	0.4200
3.	**CC**	**0.26**	**0.18**	**0.0005**
4.	**CCC**	**0.26**	**0.18**	**0.0100**
5.	**CCCG**	**0.26**	**0.18**	**0.0400**
6.	**CCG**	**0.26**	**0.18**	**0.0010**
7.	**CCGC**	**0.29**	**0.18**	**0.0100**
8.	CCGG	0.25	0.19	0.5300
9.	CCGT	0.26	0.19	0.3600
10.	CCGTT	0.21	0.19	0.5900
11.	CGT	0.26	0.18	0.1800
12.	CGCG	0.19	0.18	0.5200
13.	CGCGC	0.19	0.18	0.8800
14.	**CGCGT**	**0.17**	**0.20**	**0.0200**
15.	CGCGTG	0.17	0.19	0.0700
16.	**CGG**	**0.26**	**0.18**	**0.0020**
17.	**CGGC**	**0.32**	**0.18**	**0.0004**
18.	CGGT	0.25	0.19	0.3400
19.	CGTG	0.17	0.20	0.0700
20.	CGTT	0.19	0.18	0.5700

S.No	Motif	Median mTLR9 activity of ODNs with the motif	Median mTLR9 activity of ODNs without the motif	*p*-value
21.	CGTTC	0.22	0.18	0.1000
22.	CTG	0.18	0.19	0.2000
23.	CTGT	0.17	0.19	0.2100
24.	GAC	0.20	0.18	0.2400
25.	GAG	0.19	0.19	0.8100
26.	**GCC**	**0.26**	**0.18**	0.0400
27.	GCCG	0.25	0.18	0.0800
28.	GCGC	0.18	0.18	0.3700
29.	GCGCG	0.18	0.19	0.2600
30.	GCGCGT	0.18	0.19	0.2400
31.	GCGCT	0.18	0.19	0.3400
32.	**GCGG**	**0.27**	**0.18**	0.0040
33.	GCGT	0.18	0.20	0.3600
34.	GCGTC	0.18	0.19	0.3100
35.	GCGTG	0.18	0.19	0.1500
36.	**GG**	**0.26**	**0.18**	0.0005
37.	**GGC**	**0.30**	**0.18**	0.0002
38.	GGCC	0.17	0.19	0.9100
39.	**GGCG**	**0.31**	**0.18**	0.0006
40.	**GGG**	**0.28**	**0.18**	0.0100
41.	GGT	0.26	0.18	0.2300
42.	GTC	0.18	0.20	0.0700
43.	GTCG	0.18	0.19	0.1900
44.	GTCGC	0.18	0.19	0.1300
45.	GTCT	0.18	0.19	0.1000
46.	GTG	0.18	0.19	0.1100
47.	GTGC	0.18	0.19	0.1400
48.	GTGCG	0.19	0.19	0.8200
49.	GTGT	0.18	0.19	0.1100

S.No	Motif	Median mTLR9 activity of ODNs with the motif	Median mTLR9 activity of ODNs without the motif	*p*-value
50.	GTTC	0.20	0.18	0.6600
51.	TCC	0.26	0.18	0.0600
52.	TCCG	0.26	0.18	0.1100
53.	TCGCG	0.18	0.19	0.2500
54.	TCGCGC	0.18	0.19	0.3800
55.	**TCGG**	**0.27**	**0.18**	**0.0400**
56.	**TCT**	**0.17**	**0.20**	**0.0300**
57.	**TGC**	**0.17**	**0.24**	**0.0050**
58.	TGCG	0.18	0.19	0.3900
59.	TGCGC	0.18	0.19	0.0700
60.	TGCGCG	0.19	0.19	0.1400
61.	TGG	0.31	0.19	0.0090
62.	**TGT**	**0.18**	**0.22**	**0.0100**
63.	TGTC	0.17	0.19	0.0800
64.	TGTG	0.18	0.19	0.3500
65.	TTC	0.19	0.18	0.1800
66.	**TTGG**	**0.34**	**0.18**	**0.0060**
67.	TTT	0.19	0.18	0.2400
