# Supplementary material for: Prediction of novel mouse TLR9 agonists using a random forest approach
Source: BMC Mol Cell Biol. 2019 Dec 20;20(Suppl 2):56. doi: 10.1186/s12860-019-0241-0 (PMC6924143; doi:10.1186/s12860-019-0241-0)
Supplement: Supplementary file 3 — Additional file 3. The effect of ODN motif occurrences on the median mTLR9 activity in the high activity group. The median RAW-Blue activity for all the ODNs in the high activity group was 0.53. Increase or decrease in the median activity values due to the presence of a motif are coloured in green and red, respectively, with statistically significant values in bold. The significance threshold was set at p value < 0.05. The motifs are arranged in alphabetical order. [file 12860_2019_241_MOESM3_ESM.pdf]

Prediction of novel mouse TLR9 agonists using random forest approach

Additional File 3: The effect on the median mTLR9 activity on ODNs in the high activity group due to the presence or absence of a motif. The median mTLR9 activity for all the ODNs in the high activity group was 0.53. Increase or decrease in the median activity values due to the presence of a motif are coloured green and red, respectively, with statistically significant values in bold. The significance threshold was set at p value < 0.05. The motifs are arranged in the alphabetical order.

S.No	Motif	Median mTLR9 activity of ODNs with the motif	Median mTLR9 activity of ODNs without the motif	*p*-value
1.	**AG**	**0.47**	**0.55**	**0.04**
2.	**AGT**	**0.45**	**0.55**	**0.02**
3.	CC	0.53	0.53	0.75
4.	**CCC**	**0.51**	**0.54**	**0.82**
5.	**CCCG**	**0.51**	**0.54**	**0.78**
6.	CCG	0.53	0.53	0.82
7.	**CCGC**	**0.51**	**0.55**	**0.07**
8.	CCGG	0.53	0.52	0.63
9.	CCGT	0.51	0.54	0.70
10.	CCGTT	0.51	0.54	0.95
11.	CGT	0.55	0.52	0.81
12.	CGCG	0.51	0.56	0.26
13.	CGCGC	0.50	0.53	0.47
14.	CGCGT	0.55	0.52	0.83
15.	**CGCGTG**	**0.42**	**0.55**	**0.0002**
16.	CGG	0.53	0.53	0.94
17.	CGGC	0.56	0.52	0.16
18.	CGGT	0.50	0.54	0.22
19.	CGTG	0.52	0.54	0.28
20.	**CGTT**	**0.56**	**0.47**	**0.002**

S.No	Motif	Median mTLR9 activity of ODNs with the motif	Median mTLR9 activity of ODNs without the motif	*p*-value
21.	**CGTTC**	**0.65**	**0.50**	**0.0002**
22.	CTG	0.54	0.52	0.62
23.	CTGT	0.56	0.52	0.17
24.	GAC	0.55	0.51	0.35
25.	GAG	0.48	0.54	0.17
26.	GCC	0.54	0.53	0.59
27.	GCCG	0.53	0.53	0.98
28.	GCGC	0.51	0.55	0.16
29.	GCGCG	0.51	0.53	0.67
30.	GCGCGT	0.46	0.53	0.32
31.	GCGCT	0.52	0.53	0.68
32.	GCGG	0.51	0.54	0.35
33.	**GCGT**	**0.57**	**0.51**	**0.43**
34.	GCGTC	0.49	0.54	0.17
35.	**GCGTG**	**0.42**	**0.55**	**0.0004**
36.	GG	0.53	0.53	0.75
37.	GGC	0.55	0.52	0.14
38.	GGCC	0.52	0.53	0.29
39.	GGCG	0.56	0.52	0.55
40.	GGG	0.56	0.52	0.51
41.	GGT	0.51	0.53	0.37
42.	GTC	0.52	0.53	0.22
43.	GTCG	0.52	0.53	0.94
44.	GTCGC	0.52	0.53	0.92
45.	GTCT	0.52	0.54	1.0
46.	GTG	0.52	0.54	0.28
47.	GTGC	0.52	0.54	0.28
48.	GTGCG	0.52	0.53	0.38
49.	GTGT	0.54	0.52	0.86

S.No	Motif	Median mTLR9 activity of ODNs with the motif	Median mTLR9 activity of ODNs without the motif	*p*-value
50.	**GTTC**	**0.57**	**0.50**	0.02
51.	TCC	0.50	0.55	0.09
52.	TCCG	0.50	0.55	0.09
53.	TCGCG	0.53	0.53	0.87
54.	TCGCGC	0.60	0.54	0.63
55.	TCGG	0.59	0.52	0.07
56.	TCT	0.52	0.54	0.64
57.	TGC	0.52	0.54	0.82
58.	TGCG	0.54	0.53	0.86
59.	TGCGC	0.52	0.53	0.47
60.	TGCGCG	0.49	0.53	0.52
61.	TGG	0.55	0.52	0.37
62.	TGT	0.52	0.54	0.68
63.	TGTC	0.57	0.52	0.17
64.	TGTG	0.57	0.52	1.00
65.	TTC	0.55	0.50	0.09
66.	TTGG	0.56	0.52	0.37
67.	TTT	0.55	0.52	0.80
